# Exacerbation of Cerebellar Symptoms in Spinocerebellar Ataxia Induced by Lamotrigine: A Case Report

**DOI:** 10.7759/cureus.77448

**Published:** 2025-01-14

**Authors:** Koji Hayashi, Rina Izumi, Asuka Suzuki, Yuka Nakaya, Mamiko Sato, Toyoaki Miura, Kouji Hayashi, Yasutaka Kobayashi

**Affiliations:** 1 Department of Rehabilitation Medicine, Fukui General Hospital, Fukui, JPN; 2 Graduate School of Health Science, Fukui Health Science University, Fukui, JPN

**Keywords:** cerebellar-ataxia, lamotrigine, lamotrigine toxicity, neurological side effects, spinocerebellar ataxia

## Abstract

We describe a case of exacerbation of cerebellar symptoms following lamotrigine (LTG) treatment in a patient with the pure cerebellar type of spinocerebellar ataxia (SCA). A 16-year-old female, initially diagnosed with cerebellitis, developed progressive cerebellar ataxia despite treatment. At age 20, she was diagnosed with cerebellitis sequelae and tested positive for anti-NH_2_-terminal of α-enolase (anti-NAE) antibodies, leading to a diagnosis of cerebellar ataxia-type Hashimoto's encephalopathy. Steroid therapy provided minimal benefit, and her ataxic symptoms worsened. At age 28, a neurological examination revealed multiple cerebellar signs and significant cerebral and brainstem atrophy on magnetic resonance imaging. Due to persistent symptom progression, she was diagnosed with pure cerebellar SCA at age 30. At age 33, LTG, initiated for a mood disorder, exacerbated her cerebellar symptoms, including a new-onset tremor, downbeat nystagmus, and gait disturbance. Symptom improvement was observed following LTG dose reduction and discontinuation, suggesting LTG's influence on cerebellar function. This report discusses the effects of LTG on neurological symptoms and highlights the potential neurological side effects of LTG in a patient with progressive cerebellar ataxia, emphasizing the importance of careful medication management in neurological disorders.

## Introduction

Spinocerebellar ataxias (SCA) are a group of hereditary progressive neurodegenerative disorders characterized by ataxia, primarily due to the gradual degeneration of the cerebellum [1]. Other regions, such as the brainstem, may also be involved [1]. A systematic review estimates the global prevalence of SCA at approximately three per 100,000 people, with notable regional variations [1,2]. The core symptom triad of SCA includes gait ataxia and incoordination, nystagmus or other visual disturbances, and dysarthria [1]. In specific forms of SCA, patients may also present with additional features, such as pyramidal and extrapyramidal signs, ophthalmoplegia, and cognitive impairment [1].

Several autosomal dominant cerebellar ataxias (ADCA) have been identified. ADCA type 1 refers to cerebellar ataxia accompanied by a range of additional features, encompassing conditions like SCA1-4, 8, 10, 12-14, 15, 17-22, 25, 27, 28, 31, 32, 34-37, 38, 42-44, 46, and 47, as well as DNMT1-related ataxia and DRPLA [1]. ADCA type 2 is characterized by cerebellar ataxia with pigmentary macular degeneration and consists solely of SCA7 [1]. ADCA type 3 represents "pure" cerebellar ataxia, including SCA5, 6, 11, 23, 26, 30, 37, 41, and 45 [1]. Additionally, many patients with SCA experience psychiatric symptoms, such as depression, cognitive decline, impulsive and compulsive behaviors, anxiety, fatigue, and sleep disturbances [3].

Lamotrigine (LTG), a well-known anticonvulsant, is effective in preventing mood episodes in adults with bipolar I disorder. Its action in bipolar disorder may involve blocking sodium and calcium channels in neurons, helping stabilize cell membranes [4]. In two large 18-month randomized, double-blind trials, LTG monotherapy significantly delayed the need for additional treatments (medication or electroconvulsive therapy) for new mood episodes compared to placebo. LTG particularly extended the time before intervention for depressive episodes in both recently manic/hypomanic and recently depressed patients [4]. While pooled data indicated LTG's effectiveness in delaying mania/hypomania episodes, lithium outperformed LTG in this regard [4]. In addition, LTG has been reported to provide significant relief from gait disturbance in patients with SCA type 3 (SCA3), also known as Machado-Joseph disease (MJD), who have early-stage ataxia [5]. To our knowledge, there are no other reports on the effects of LTG in SCA subtypes other than SCA3. In this report, we describe a case of SCA in which the patient developed tremors, along with worsening gait disturbances and nystagmus, during LTG treatment.

## Case presentation

**Figure 1:**
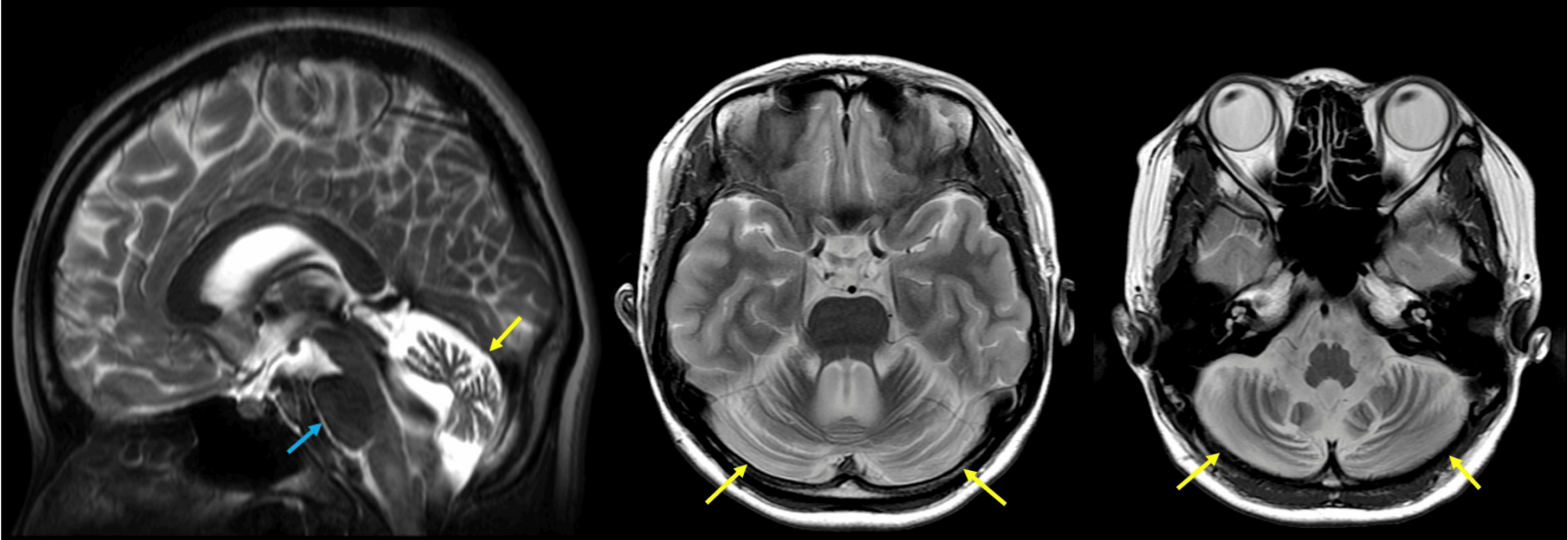
The result of brain magnetic resonance imaging (MRI) at 28 years old. T2-weighted brain MRI shows significant cerebellar atrophy (yellow arrows) and mild brainstem atrophy (blue arrow).

A 16-year-old previously healthy woman developed a headache and fever, visited another hospital, and was diagnosed with cerebellitis. She had no family history of neuromuscular disease. Although the exact course of the disease is unclear, her cerebellar symptoms gradually worsened even after she returned home. At the age of 20, she visited another hospital due to worsening symptoms. She was diagnosed with cerebellitis sequelae, characterized by tremors and an inability to perform tandem gait. In the same year, she tested positive for anti-NH_2_-terminal of α-enolase (anti-NAE) antibodies, leading to a diagnosis of cerebellar ataxia-type Hashimoto's encephalopathy. Although steroid therapy was administered, its effectiveness was minimal, and her ataxic symptoms progressively worsened. At the age of 28, she visited our hospital for rehabilitation. Neurological examination revealed gaze-induced nystagmus, dysarthria, hypotonia, hyperreflexia in the patellar tendon, dysmetria on the finger-nose-finger test, and truncal ataxia. Dysphagia, tremors (including rest and postural tremors), and bladder or rectal disorders were not observed. Romberg's sign and Babinski's were negative. Brain magnetic resonance imaging (MRI) revealed significant atrophy of the cerebrum and brainstem (Figure 1). Due to the continued progression of symptoms, she was ultimately diagnosed with the pure cerebellar type of SCA at the age of 30. She declined genetic testing.

**Figure 2:**
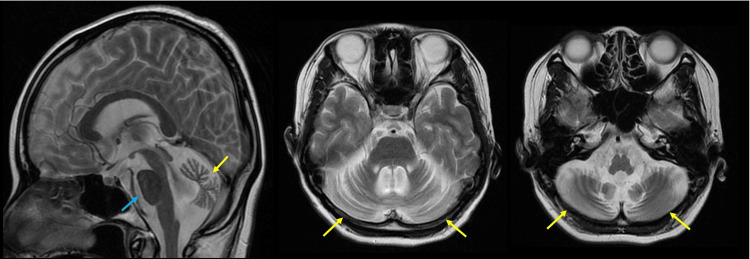
The result of brain magnetic resonance imaging (MRI) at 32 years old. T2-weighted brain MRI shows significant cerebellar atrophy (yellow arrows) and mild brainstem atrophy (blue arrow), with minimal changes compared to the results at 28 years old.

At the age of 32, she developed slight downbeat nystagmus. Brain MRI again revealed significant atrophy of the cerebrum and brainstem, with almost no change compared to the results at the age of 28 (Figure 2). At 33 years old, LTG (10 mg every other day) was prescribed for mood disorders and was gradually titrated up to 200 mg/day. Unfortunately, blood levels of LTG were not measured. During this dose increase, she developed a previously unobserved tremor affecting her neck and trunk, along with exacerbation of downbeat nystagmus and gait disturbance. The limb tremors were fine, and the cervical tremor presented as a no-no tremor. Before LTG administration, she could walk independently, but after taking LTG, she required assistance. When the LTG dose was reduced to 100 mg/day, the tremor subsided, and she was able to walk with support. After LTG was discontinued, the gait, tremor, and downbeat nystagmus returned to their pre-administration levels.

## Discussion

We describe a case of a developed tremor affecting the neck and trunk and worsened gait disturbance and nystagmus in a patient with SCA during LTG treatment. Although brain MRI revealed atrophy of both the brainstem and cerebellum, her clinical findings were limited to cerebellar symptoms, suggesting a pure cerebellar type of SCA. Anti-NAE antibodies are widely recognized as markers for Hashimoto's encephalopathy; however, some patients present with insidious onsets, such as spinocerebellar degeneration (SCD) [6]. In this case, steroids were administered due to the presence of anti-NAE antibodies. However, the response to treatment was limited, and the symptoms continued to progress steadily, supporting the diagnosis of SCA. LTG had been prescribed by a psychiatrist for bipolar disorder, which led to the development of downbeat nystagmus, tremors in the head and body, and an exacerbation of gait disturbance that required the use of a wheelchair. After LTG was discontinued, the gait, tremor, and downbeat nystagmus returned to their pre-administration levels.

A review of LTG's neurological side effects, particularly in movement disorders, summarized that LTG may be associated with tics, dyskinesias, myoclonus, parkinsonism, dystonia, stuttering, akathisia, cerebellar syndromes, hypertonia, and dyskinesia [7]. Concerning cerebellar symptoms, Hajiali *et al.* reported a case involving a 26-year-old female with a history of bipolar mood disorder but no neurodegenerative disease. After overdosing on LTG (200 tablets of 200 mg each), she developed symptoms including weakness, fatigue, drowsiness, agitation, tachycardia, lethargy, dehydration, slurred speech, and cerebellar manifestations such as nystagmus and ataxia [8]. Her condition returned to normal as LTG levels decreased. Additionally, Moreira *et al. *described four cases of neurological withdrawal symptoms related to combined LTG and valproic acid therapy [9]. Three of these cases experienced cerebellar or related symptoms, including opsoclonus, ataxia, nystagmus, vertigo, speech and gait disturbances, and disabling tremors [9]. All three cases showed symptom improvement after a reduction in LTG dosage. It is well known that valproic acid raises blood levels of LTG [10], and one other reported case involved the onset of symptoms following LTG overdose [8]. Based on these findings, previous reports suggest that excessively elevated blood concentrations of LTG may contribute to the development of cerebellar symptoms.

In our case, the patient was prescribed the usual dose of LTG. Since cerebellar symptoms are the main features of SCA, downbeat nystagmus and truncal ataxia may have appeared even at an appropriate LTG dosage. Similar to previous reports, these symptoms disappeared once the LTG dose was reduced, suggesting that LTG may influence cerebellar symptoms through certain mechanisms.

Although our case did not improve and instead experienced worsening cerebellar symptoms with LTG treatment, there is evidence suggesting that LTG has neuroprotective effects. Kremer *et al. *reported significant symptomatic improvement and a tendency toward reduced chorea in patients with Huntington's disease after 30 months of LTG treatment [11]. Additionally, Liu *et al. *reported a case series of patients with MJD treated with LTG that showed significant improvement in gait disturbance [5]. The authors demonstrated that LTG decreased mutant ataxin-3 levels in vitro, concluding that LTG elicits a positive response in MJD patients with mild truncal ataxia through its pharmacological action involving the downregulation of mutant ataxin-3 [5].

In our case, although genetic tests were not conducted, we clinically diagnosed the patient with the pure cerebellar type of SCA, as her symptoms were different from those of MJD. Thus, the effect of LTG differed from that observed in MJD, despite both being types of SCA, and it is possible that LTG contributed to worsening rather than improving her walking disorder. Additionally, previous research has shown that high or excessive blood concentrations of LTG can exacerbate cerebellar symptoms [8,9]. This suggests that, except in certain specific neurodegenerative diseases, LTG may worsen cerebellar symptoms. The primary symptoms of pure cerebellar type of SCA include ataxia and nystagmus, and LTG may have a tendency to induce or exacerbate these symptoms, even at standard dosages.

As a limitation, we did not measure the blood concentration of LTG. Our case did not involve the use of valproic acid or other medications known to interact with LTG. However, we were unable to exclude other factors that could increase the concentration of LTG, even at a normal dosage. If we had measured the blood concentration of LTG, we could have provided a more convincing presentation.

## Conclusions

SCA is often associated with mood disorders, making their management an essential aspect of care. LTG is one of the key medications for controlling mood disorders and epilepsy; however, it may exacerbate cerebellar symptoms. While the efficacy of LTG has been reported in certain neurological conditions, our case demonstrated a significant worsening of cerebellar symptoms during LTG administration. Further research is needed to elucidate the mechanism by which LTG aggravates cerebellar symptoms.

## References

[1] Sullivan R, Yau WY, O'Connor E, Houlden H (2019). Spinocerebellar ataxia: an update. J Neurol.

[2] Ruano L, Melo C, Silva MC, Coutinho P (2014). The global epidemiology of hereditary ataxia and spastic paraplegia: a systematic review of prevalence studies. Neuroepidemiology.

[3] Lin CR, Kuo SH, Opal P (2024). Cognitive, emotional, and other non-motor symptoms of spinocerebellar ataxias. Curr Neurol Neurosci Rep.

[4] Goldsmith DR, Wagstaff AJ, Ibbotson T, Perry CM (2003). Lamotrigine: a review of its use in bipolar disorder. Drugs.

[5] Liu CS, Hsu HM, Cheng WL, Hsieh M (2005). Clinical and molecular events in patients with Machado-Joseph disease under lamotrigine therapy. Acta Neurol Scand.

[6] Matsunaga A, Ikawa M, Yoneda M (2024). Expanding clinical spectrum from Hashimoto's encephalopathy to anti-NAE antibody-associated disorders (NAEAD). Clin Exp Neuroimmunol.

[7] Rissardo JP, Fornari Caprara AL (2021). Lamotrigine-associated movement disorder: a literature review. Neurol India.

[8] Hajiali F, Nassiri-Asl M (2015). Report of severe menorrhagia following the maximum amount of lamotrigine overdose. Iran J Pharm Res.

[9] Moreira B, Thomé-Souza S, Valente K (2007). Late side-effects of valproate and lamotrigine. J Epilepsy Clin Neurophysiol.

[10] Yamamoto Y, Usui N, Kagawa Y, Imai K (2024). Time-course changes in lamotrigine concentration after addition of valproate and the safety and long-term tolerability of lamotrigine-valproate combination therapy. Biol Pharm Bull.

[11] Kremer B, Clark CM, Almqvist EW (1999). Influence of lamotrigine on progression of early Huntington disease: a randomized clinical trial. Neurology.

